# A Cure for Love Melancholy

**Published:** 1871-03

**Authors:** 


### A CURE FOR LOVE MELANCHOLY.

Dr. James Ferrard, of Oxford, in 1640, published the following treatment for *love melancholy:* "The regimen, or order of diet, in cure of love melancholy, differs not at all from that which is to be observed in the prevention of it, save only that it ought to be somewhat more humectative and less refrigative; not forgetting, in the meantime, those meats that by some certaine occult properties they have in them, are found to be very good for those that are sick of this disease; as the turtle-dove, the heart of a wolfe, young owles taken and boyled in the juyce of marioram, the flesh of rats, and the like. And if the party be fallen away in his body, and is now grown very thin and dry, you must then prescribe him the same order of diet, according to Avicen, as you doe to those that are hecticall."

